# Front‐line staff perspectives on a caring culture in Chinese hospitals: Validation of a Chinese version of the Culture of Care Barometer

**DOI:** 10.1111/jonm.13657

**Published:** 2022-05-31

**Authors:** Liying Ying, Joanne M. Fitzpatrick, Julia Philippou, Yaping Zhang, Trevor Murrells, Anne Marie Rafferty

**Affiliations:** ^1^ Department of Nursing The Second Affiliated Hospital of Zhejiang University School of Medicine Hangzhou China; ^2^ Florence Nightingale Faculty of Nursing, Midwifery and Palliative Care King's College London London UK; ^3^ Department of Nursing, School of Medicine Zhejiang University Hangzhou China; ^4^ General Office of the Administration Zhejiang Provincial People's Hospital Hangzhou China

**Keywords:** caring culture, China, Culture of Care Barometer, measurement, validation

## Abstract

**Aims:**

The aim of this study is to examine the psychometric properties of the Chinese version of the Culture of Care Barometer in health care organizations.

**Background:**

There is a lack of tools to gauge the caring culture in Chinese hospitals. The Culture of Care Barometer is a psychometrically sound measure for caring culture developed in Western settings.

**Methods:**

This study was guided by Sousa and Rojjanasrira's methodological approach. A total of 2365 staff were recruited from two tertiary hospitals. The Barometer was administered with the Hospital Culture Evaluation Index and Minnesota Satisfaction Questionnaire.

**Results:**

The content validity index was calculated as 0.99. The goodness‐of‐fit indices, apart from the model chi‐square, which was statistically significant, all exceeded established thresholds for adequate fit. The internal consistency was very satisfactory. Pearson's correlation indicated that the tool has good concurrent and convergent validity.

**Conclusions:**

The Barometer is a reliable and valid instrument to assess front‐line staff perspectives on a caring culture in Chinese hospitals.

**Implications for Nursing Management:**

Nursing managers can use the Barometer to gauge the caring culture in China. Tailored interventions can be designed to address specific domains, and additional support can be provided to more vulnerable departments or staff groups.

## 1 BACKGROUND

The culture of care in health care organizations refers to the contextual structures that influence how individuals make and express meaning in the care of patients through creating dominant values, norms, and beliefs (Rytterstrom et al., 2013). The culture of care describes the part of culture that follows the common good as its guiding principle (Salmela et al., 2017). The concept of caring in health care systems represents a broader meaning than ‘patient‐’ or ‘person‐’centred care, in that it recognizes the need for staff themselves to work in an enriched environment if they are to create such an environment for patients and their carers (Rafferty et al., 2015). A caring culture is positively related to interprofessional collaboration, employee well‐being, job performance and organizational commitment (Luthans et al., 2008; Wei et al., 2019). Evidence has demonstrated that the well‐being of staff is closely linked to the well‐being of patients, and staff commitment is a key predictor of a wide range of outcomes in health care organizations (Powell et al., 2014).

To foster a caring environment, it is a paramount to assess the culture of care from the perspective of staff. An initial analysis of the literature revealed a lack of instruments for measuring ‘care cultures’ as distinct from organizational culture or patient safety culture (Hesselink et al., 2013). The attributes of care culture for patients or staff are also not captured in the extant measures for quality and performance in health care organizations (Hesselink et al., 2013). The Culture of Care Barometer (CoCB) tool was thus developed to gauge the different attributes of care culture perceived by health care staff (Rafferty et al., 2017). The CoCB comprises 30 items organized into four domains: organizational values, team support, relationships with colleagues and job constraints. The CoCB can act as a ‘diagnostic’ measurement to assess the culture of care of health care organizations, and as a ‘dialogic’ tool, designed to prompt reflection on the underlying issues involved in creating a caring culture (Rafferty et al., 2017). The tool has been tested with nurses and other health care providers in England and indicated good reliability and validity (Rafferty et al., 2017). Further studies, to adapt and test the tool in other countries, are needed to enable validation of the tool in a global context.

Given its significance for care quality and staff engagement, the nurturing of a caring culture is relevant to all health care systems globally. This includes China where in recent years, promoting patient safety and reducing the turnover rate of nurses are government priorities. According to an analysis of the database of China Patient Safety Incidents Reporting System, the number of incident reports increased from 815 in 2012 to 8088 in 2017 (Gao et al., 2019). As well as negative consequences for patients, unsafe practice including practice errors has also triggered hospital violence against staff in China (Jia et al., 2014). It is known that patient safety is positively associated with the work environment, directly and indirectly (Sun et al., 2018). In addition, it was reported in another study that 36% (279/778) of experienced nurses who were employed for at least 5 years in China had a high‐level of turnover intention, measured by Farh's Turnover Intention Scale (Wan et al., 2018). Scale scores were negatively associated with work environment and mediated by work engagement. It is imperative therefore to create enriched caring environments in health care organizations in China to improve care quality, staff retention and well‐being.

The purpose of this research is to test and implement the Chinese version of the CoCB in two health care organizations in China. Specifically, the objectives are as follows: (a) to translate the CoCB into Chinese, (b) to determine the psychometric properties of the CoCB with a sample of Chinese nurses and physicians and (c) to assess front‐line staff perspectives on caring culture in Chinese hospitals using the CoCB. This is the first Chinese version of an instrument that assesses caring culture in health care systems. The findings of this cross‐sectional study will enable health care organizations and researchers to develop interventions that aim to improve the culture of care. It is hoped that improving patient safety and reducing nursing staff turnover could also be achieved.

## 2 METHODS

### 2.1 Study design

Sousa and Rojjanasrirat's (2011) methodological approach for translation, adaptation and validation of instruments for use in cross‐cultural health care research was adopted to guide the study. Three stages were undertaken, namely, cross‐cultural translation, pilot testing and full psychometric testing (Sousa & Rojjanasrirat, 2011). The STROBE checklist for observational research has been followed for presenting the research (see File S1 in the supporting information).

#### 2.1.1 Cross‐culture translation

The CoCB was translated into Chinese following the standard procedures including forward translation, comparison of the two translated versions, blind back‐translation and comparison of the two back‐translated versions with the original tool (Sousa & Rojjanasrirat, 2011). First, a native Chinese bilingual nursing postdoctoral fellow and a professional translator with over 5 years of experience in translation made the forward translation of the CoCB into simplified Chinese. Some idiomatic expressions in the scale like ‘trust, line manager’ were changed to ‘hospital, direct supervisor’ in discussion with the authors of the CoCB. Second, a second nursing postdoctoral fellow compared the two translated versions. Any ambiguities and discrepancies were discussed and resolved by a committee which comprised the two translators, three doctoral nursing graduates and a clinical nurse. Third, the preliminary translated version of the CoCB was back‐translated into English by a Chinese bilingual doctoral nursing graduate and a professional translator with 12 years' experience. Finally, a comparison between the back‐translations of the CoCB and the original CoCB was made by a committee which comprised the other members of the research team and all involved individuals (the two translators, two fellows, three nursing graduates and one clinical nurse). The similarity of the items regarding wording, sentence structure, meaning and relevance was evaluated and discussed. This process resulted in the Chinese version of the CoCB (CoCB‐C) that was digitalized and tested.

#### 2.1.2 Pilot testing

Pilot testing of the CoCB was conducted using expert review and a small‐scale cross‐sectional survey. A panel of six experts from two tertiary hospitals (Sousa & Rojjanasrirat, 2011), including a vice president, two heads of department, a nursing director, a nursing manager and an associate professor of medicine in the research management department, were invited to identify the semantic equivalence and content validity of the CoCB‐C. The comments were also solicited. Tertiary hospitals in China refer to the comprehensive or general hospitals at city, provincial or national level with a bed capacity exceeding 500. Each panel member rated all individual items for semantic equivalence using a dichotomous scale (yes or no) (Sousa & Rojjanasrirat, 2011). Any item or instruction that is found to be unclear by at least 20% of the participants must be revised and re‐evaluated (Sousa & Rojjanasrirat, 2011). Each item was rated as ‘clear’ by more than 80% of the panel members (range, 83.3%–100%). The inter‐rater agreement was 96.7%.

The experts were also invited to evaluate the content validity of the CoCB‐C. Each expert rated the content relevance of each CoCB‐C item in measuring the culture of care in the hospital in China. An ascending 4‐pointing Likert scale was used. The content validity index at the item level (i.e., the percentage of experts who rated the items as 3 or above on the Likert scale) and at the scale level (i.e., the mean percentage of items that were rated as 3 or above on the Likert scale) in the first round was calculated as 0.83–1.00 and 0.99 respectively, which indicates good content validity for the CoCB‐C (Polit et al., 2007). A few comments about wording and sequence of items were proposed by experts. Minor modifications were made after intensive discussion in the translation committee and research team. There were no additions or deletions of items. This process resulted in the final Chinese version of the CoCB (see Appendix).

A total sample of 20 (Sousa & Rojjanasrirat, 2011) registered nurses and physicians working for at least 6 months in the surgical department of a tertiary hospital in Hangzhou city, China, was recruited through a convenience sampling approach to complete the CoCB‐C. A total of nine nurses and 11 surgeons participated; they were also asked to rate the tool items and instructions for clarity using a dichotomous scale (clear or unclear). No modifications were needed. Cronbach's alpha coefficient was 0.95, indicating very good internal consistency (DeVellis, 2016).

#### 2.1.3 Psychometric testing

A convenience sample of 2365 staff was recruited from two tertiary hospitals in Hangzhou, Zhejiang Province, China, between November and December 2019.The trained local collaborators, including a surgeon and an assistant to the director of the hospital, administered the invitation letters, the participant information sheets and the link of the survey to the staff using the official internal online working groups. The survey was conducted anonymously through a secure online survey system called Questionnaire Star. Inclusion criteria were (i) registered nurses, medical doctors, dentists, allied health professionals, administrative and clerical staff, estates and facilities staff; (ii) working for at least 6 months in the participating hospitals; and (iii) Chinese ethnicity. Staff who had come to the hospital for temporary training were excluded. The ‘Rule of 10 subjects per item of the instrument’ was used to guide the sample size planning for conducting confirmatory factor analysis (CFA), with a target of at least 300 participants (Gonzalez & Griffin, 2001; Sousa & Rojjanasrirat, 2011). All 4738 staff received the study information from the trained local collaborators. Those eligible for inclusion were invited to participate, and a total of 2672 staff completed the survey of whom 2365 were eligible for statistical analysis. Records with potential response biases, for example, records with the same answer option for all items of the scale, or a response time of less than 3 min (mean = 8.00 ± 17.12), or not meeting the target criteria, were excluded.

### 2.2 Instruments

#### 2.2.1 CoCB (Chinese version; CoCB‐C)

The original English version of the CoCB measures the cultural attributes of environments in which care is delivered. It is a 30‐item self‐reported questionnaire. Responses are measured on a 5‐point Likert scale from ‘not at all’ to ‘fully agree’, with higher scores representing a better caring culture of care in the settings. Cronbach's alphas of 0.70 to 0.93 were reported in the original study (Rafferty et al., 2017). The exploratory factor analysis of the CoCB provides evidence of good construct validity (Rafferty et al., 2017).

#### 2.2.2 Hospital culture evaluation index (HCEI)

The 32‐item HCEI was administered to health care workers to assess the organizational culture of the hospitals (Chang & Cheng, 2009). This instrument includes four aspects of hospital culture, namely, the material culture, behavioural culture, institutional culture and spiritual culture. Responses are measured on a ‘1–5’ Likert scale from ‘fully disagree’ to ‘fully agree’, with higher scores representing a more positive hospital culture. It has been used and validated in the Chinese hospital, which indicated good internal consistency (Cronbach's *α* = 0.81) and good validity (content validity index 0.78) (Xi et al., 2016). The HCEI is a reliable and valid measure having similar constructs to the CoCB‐C. The results of this scale were then used to conduct convergent validity testing of the new tool.

#### 2.2.3 Minnesota Satisfaction Questionnaire (MSQ)‐short form

The 20‐item MSQ short form (Weiss, Dawis & England, 1967) was used in this study to measure the job satisfaction of employees. It has three dimensions: intrinsic job satisfaction, extrinsic job satisfaction and general job satisfaction. A 5‐point Likert scale from ‘very dissatisfied’ to ‘very satisfied’ is used to rate each statement, with higher scores representing better job satisfaction. Cronbach's alphas of 0.84 to 0.93 were reported, indicating good internal consistency of the tool (Weiss et al., 1967). The Chinese version of the MSQ short form is presented on the website of the University of Minnesota (http://vpr.psych.umn.edu/instruments/msq-minnesota-satisfaction-questionnaire). It has been used in hospitals in China (Jiang et al., 2018; Xi et al., 2016) and with good reliability and validity; Xi et al. (2016) reported a Cronbach's *α* of 0.92, and the value of Kaiser–Meyer–Olkin was 0.92. The MSQ is a well‐used instrument and is stable over the time. It assesses a concept that is often related to the culture of care in the hospitals. The findings of this scale were then used to conduct concurrent validity testing of the CoCB‐C.

### 2.3 Data analysis

Descriptive statistics were used to summarize the participants' characteristics. The attributes of reliability assessed for the CoCB‐C were internal consistency and item‐to‐total correlations. Cronbach's alpha values of 0.70 or greater indicate adequate internal consistency (DeVellis, 2016).

The percentages of participants scoring the minimum (floor) and maximum (ceiling) possible scores were calculated. The important floor or ceiling effects were defined as more than 15% of participants achieving the lowest or highest score, respectively (Terwee et al., 2007). The initial discrimination ability of CoCB‐C was examined by contrasted‐groups validity (Molassiotis et al., 2007). High and low score groups of CoCB‐C were taken from upper and lower 27% of participants, respectively (Hingorjo & Jaleel, 2012). Based on the literature, we anticipated that responses in high score group would show significantly higher positive hospital culture and better job satisfaction than those in low score groups (Luthans et al., 2008; Rafferty et al., 2015).

CFA was used to test whether the data confirm the proposed four‐factor CoCB model. The CoCB‐C items were treated as ordinal variables in the analysis due to the distribution of item scores, many of which were skewed towards the upper end of the 5‐point scale. Goodness‐of‐fit was examined using the comparative fit index (values >.90 indicating acceptable model fit), the Tucker–Lewis index (values >.90 indicating acceptable model fit), the root‐mean‐square error of approximation (values <.08 indicating adequate model fit) and the standardized root‐mean‐square residual (values <.08 indicating reasonably good model fit) (Brown, 2015).

Concurrent validity (i.e., correlations between different instruments designed to assess two presumably related constructs) was examined by hypothesis testing. The hypothesis was that staff working in a more caring culture (CoCB‐C) would have better job satisfaction (Luthans et al., 2008). Convergent validity (i.e., correlations between different instruments designed to assess a common construct) of the CoCB‐C was examined using its correlation with the HCEI, which was used to measure organizational culture in hospitals. Pearson product–moment correlations were used to test all the relationships, with positive and statistically significant correlations (*p* < .05) indicating good concurrent and convergent validity.

CFA was performed using the R Lavaan package (Rosseel, 2012) and all other analyses using IBM SPSS 24.0 software (IBM Corp, 2016).

### 2.4 Ethical considerations

Ethical approval was obtained from the Ethics Review Committee of King's College London (Reference number: LRS‐18/19‐11872), and access permissions were sought from the study sites. The participants had been informed that the completion of the survey constituted consent to participate and all data collected to be used.

## 3 RESULTS

### 3.1 Participants' characteristics

The demographic characteristics of the participants are summarized in Table 1. Nearly four of five participants were female (77.3%, *n =* 1828), and 35.3% (*n =* 836) were aged 30 years or younger. One third of the participants had less than 6 years work experience. Over half of participants were nurses (58.2%, *n =* 1377), compared with just over a fifth who were medical doctors (21.1%, *n =* 498). The majority (85.7%, *n =* 2027) of participants held a bachelor or master's degree. Regarding professional title, about half of them were the junior titles (e.g., resident doctor and nurse practitioner) (45.1%, *n =* 1067). About 14.4% (*n =* 340) were team managers. Only two out of 21 hospital managers responded to the survey. Floor or ceiling effects for COCB‐C total scores were reported in Table S1. The percentages of respondents with either floor (*n =* 0, 0%) or ceiling effects (*n =* 320, 13.5%) were below the threshold of 15%.

**TABLE 1:** Participants characteristics (*n =* 2365)

	Percentage (%)		
	Hospital 1 (*n* = 2087)	Hospital 2 (*n* = 278)	Overall (*n* = 2365)
Gender			
Female	77.4	76.3	77.3
Male	22.6	23.7	22.7
Age groups, years			
19–30	34.4	42.8	35.3
31–40	35.5	41.7	36.2
41–50	21.3	12.2	20.2
51–60	8.9	3.2	8.2
Work experience, whole years			
0.5–5	32.5	37.8	33.1
6–10	24.3	28.4	24.8
11–20	19.1	25.5	19.8
>20	24.1	8.3	22.2
Staff group			
Medical doctor/dentist	19.6	32.0	21.1
Registered nursing staff	57.6	62.9	58.2
Allied health professionals	12.6	1.8	11.3
Administrative and clerical staff	6.1	3.2	5.8
Estates and facilities	4.1	0.0	3.6
Education			
High school	2.8	0.4	2.5
Associate degree	6.8	14.0	7.6
Bachelor degree	70.8	59.0	69.4
Master's degree	15.4	23.0	16.3
Doctoral degree	4.3	3.6	4.2
Professional title^a^			
Junior	44.4	50.3	45.1
Intermediate	41.2	33.1	40.2
Senior	14.4	16.5	14.7
Position			
Hospital manager	0.1	0.0	0.1
Team manager	15.0	9.4	14.4
General staff	84.9	90.6	85.5

^a^ **Junior professional titles** refer to resident doctor, nurse practitioner, pharmacist, nurse, assistant pharmacist and so on. **Intermediate professional titles** refer to attending doctor, nurse‐in‐charge, pharmacist‐in‐charge and so on. **Senior professional titles** refer to (associate) chief physician, (associate) chief nurse, (associate) chief pharmacist and so on.

### 3.2 Contrasted‐groups validity

The scores of HCEI and MSQ in the two groups of participants (upper 27% and lower 27%) were compared and were found to be significantly different (All *P* < .001), with high score group demonstrating higher positive hospital culture (HCEI, mea*n =* 158.95 vs. 115.26) and better job satisfaction (MSQ, mea*n =* 97.55 vs. 68.76), as expected.

### 3.3 CFA of CoCB‐C

The overall CFA model chi‐square statistic was significant (*χ* ^2^ = 5975.22, 399 *df*, *P* < .0001). The other goodness‐of‐fit indices, the comparative fit index (0.998), Tucker–Lewis index (0.998), root‐mean‐square approximation (0.074) and standardized root‐mean‐square residual (0.036), all exceeded the thresholds for an adequate fitting model. Taken together, these findings indicate that the data showed an acceptable fit to the original four‐factor model. As presented in Table 2, the factor loadings of the items were all greater than 0.55, indicating adequate relevance to their respective factors.

**TABLE 2:** Factor loadings of culture of care barometer (Chinese version; CoCB‐C)

Culture of care barometer (Chinese version; item no. and content)	Factor 1 (organizational values)	Factor 2 (team support)	Factor 3 (relationship with colleagues)	Factor 4 (job constraints)
(4) I am proud to work in this hospital	0.84			
(6) The hospital values the service we provide	0.85			
(7) I would recommend this hospital as a good place to work	0.83			
(13) There is strong leadership at the highest level in the hospital	0.79			
(15) Hospital managers know how things really are in the hospital	0.84			
(19) A positive culture is visible where I work	0.84			
(22) Staff successes are celebrated by the hospital	0.84			
(23) The hospital listens to staff views	0.88			
(24) I get the training and development I need	0.86			
(25) I am able to influence the hospital how things are done in the hospital	0.67			
(26) The hospital has a positive culture	0.87			
(29) I feel well informed about what is happening in the hospital	0.82			
(5) My direct supervisor treats me with respect		0.82		
(8) I feel well supported by my direct supervisor		0.87		
(9) I am able to influence the way things are done in my team		0.68		
(10) I feel part of a well‐managed team		0.85		
(11) I know who my direct supervisor is		0.57		
(12) Unacceptable behaviour is consistently tackled in the team		0.71		
(16) I feel able to ask for help when I need it		0.84		
(18) I feel supported by the team to develop my potential		0.87		
(21) My direct supervisor gives me constructive feedback		0.88		
(27) I am kept well informed about what is going on in our team		0.84		
(30) My concerns are taken seriously by my direct supervisor		0.89		
(2) I feel respected by my co‐workers			0.67	
(14) When things get difficult, I can rely on my colleagues			0.83	
(20) The people I work with are friendly			0.81	
(28) I have positive role models where I work			0.81	
(1) I have the resources I need to do a good job				0.87
(3) I have sufficient time to do my job well				0.78
(17) I know exactly what is expected of me in my job				0.61

### 3.4 Reliability of CoCB‐C

The internal consistencies (Cronbach's *α*) for the four factors were all above the required thresholds (organizational values, 0.96; team support, 0.95; relationship with colleagues, 0.86; and job constrains, 0.77). All corrected item‐to‐total correlations for the factors were greater than 0.40, indicating homogeneity with their respective factor (organizational values, 0.65 to 0.87; team support, 0.55 to 0.87; relationship with colleagues, 0.61 to 0.76; and job constraints, 0.47 to 0.74). The subscales were strongly intercorrelated (*r* = 0.75 to 0.91, all *P* < .001), giving further support to the use of the overall scale of the CoCB‐C. The overall internal consistency of the CoCB‐C was 0.82.

### 3.5 Concurrent validity and convergent validity

Significant and positive correlations were found between the overall score of CoCB‐C and scores on MSQ and HCEI, indicating a good concurrent and convergent validity. A better caring culture was associated with greater job satisfaction (*r* = 0.92, *P* < .001) and a better organizational culture (*r* = 0.88, *P* < .001). Examination of the subscales found that these relationships occurred more strongly for organizational values and team support (MSQ, *r* = 0.90, 0.89; HCEI, *r* = 0.90, 0.81) than for relationship with colleagues and job constraints (MSQ, *r* = 0.82, 0.80; HCEI, *r* = 0.76, 0.74).

## 4 DISCUSSION

This study conducted a cross‐culture translation and validation of the CoCB (Rafferty et al., 2017). This was the first Chinese version of an instrument that assesses the caring culture in health care systems from the perspective of front‐line staff. Results indicated that the CoCB‐C has a stable factor structure, reasonable model fit and high internal consistency. No notable floor and ceiling effects were detected for the CoCB‐C total score. Its strong significant correlation with the theoretically linked constructs including job satisfaction of employees and organizational culture of hospital is evidence of its concurrent and convergent validity. The findings concurred with the psychometric testing of the original version of the CoCB developed in England for health care providers (Rafferty et al., 2017). With minor revisions (e.g., wording of items to fit the context), the CoCB can be used in Chinese tertiary hospitals, yielding internationally comparable results.

The use of a strict method for validation (Sousa & Rojjanasrirat, 2011) strengthens the results from this study. A large, convenience sample of 2365 staff generated data for psychometric testing. The response rate of the two settings was uneven (59.0% vs. 23.2%). A possible reason was that one local collaborator, currently a hospital manager, was more appealing to the staff than the other surgeon collaborator. Nevertheless, our sample size exceeds the number recommended for CFA (Gonzalez & Griffin, 2001; Rosseel, 2012; Sousa & Rojjanasrirat, 2011) and is larger than the sample (*n =* 1698) used to develop the CoCB in the UK (Rafferty et al., 2017). Given the differences in the medical system and health care administration, some adjustments were made in the Chinese translation to enable front‐line staff to better understand the questionnaire. The adaptation of the items attained a high rate of agreement (96.7%) among the panel members, and each item has been rated as ‘clear’ by more than 80% of the medical staff participating in the pilot testing. Therefore, it can be stated that its semantic equivalence between the translated version and original version was achieved.

In this study, a Cronbach's alpha coefficient of 0.82 for the overall scale was obtained which exceeds the threshold of 0.70 for adequate internal consistency (DeVellis, 2016). The values obtained for the four factors were similar to the original scale (CoCB vs. CoCB‐C: *α* = 0.93, 0.93, 0.84, 0.70 vs. 0.96, 0.95, 0.86, 0.77) (Rafferty et al., 2017), so both the English and Chinese versions have good and equivalent internal consistency. Content validity was high, indicating that all items are measuring the same construct as the overall scale. Furthermore, the CoCB‐C was able to show initial discrimination between groups theoretically demonstrated as experiencing disparate hospital culture and job satisfaction.

CFA is an appropriate analytic technique when there is a previous study that specifies the item composition of theoretically meaningful latent factors (Brown, 2015). In this study, the overall chi‐square statistic of the goodness of model fit index was statistically significant (*P* < .001). The chi‐square test is known to be sensitive to large sample size (Brown, 2015); therefore, we made use of other common measures of fit. All of them were acceptable. For the factor loadings of the items, values obtained were slightly higher than those from the UK study (CoCB vs. CoCB‐C: organizational values, 0.40 to 0.84 vs. 0.67 to 0.88; team support, 0.40 to 0.87 vs. 0.57 to 0.89; relationship with colleagues, 0.56 to 0.81 vs. 0.67 to 0.83; and job constraints, 0.41 to 0.79 vs. 0.61 to 0.87) (Rafferty et al., 2017). Therefore, the results supported the generalizability of the CoCB factor structure.

A high convergent validity of the CoCB‐C was shown through its correlation with the HCEI. Although both scales measure the culture in health care organizations, the CoCB‐C emphasizes caring culture from the perspectives of staff. This is an essential but often overlooked issue in China, where the cultural value of collectivism is advocated (Voronov & Singer, 2002). Chinese people, especially health care professionals, are expected to give more consideration to the needs and interests of others than to themselves. As an example, in the context of the COVID‐19 pandemic, a heavily pregnant nurse who worked in a highly contagious environment was regarded as a self‐sacrificing hero by the state media (Zhu et al., 2021). Hence, greater attention to understanding front‐line staff perspectives and needs for a caring culture in their workplace is paramount.

Our study identified a strong positive relationship between a hospital's perceived caring culture and staff job satisfaction, which occurred more strongly on macro and meso levels than on micro level. This result echoes previous evidence about the positive effects of a good caring culture in work organizations on the job satisfaction of employees (Luthans et al., 2008). Such findings have important clinical implications in China where nearly half (735/1473) of the nurses (Liu, Zheng, et al., 2019) and 64.8% (473/730) of medical doctors (Liu, Yu, et al., 2019) were dissatisfied with their current job. As a consequence of the current COVID‐19 pandemic, health care workers have reported increased psychological distress and job stress (Lai et al., 2020; Zhan et al., 2020), which would possibly result in poorer job satisfaction and trigger turnover intention (Cai et al., 2021). Therefore, it is essential for hospital leaders and nurse managers to establish a caring culture in which care is delivered, particularly on the levels of organization and team.

The CoCB‐C can contribute to advancing research. It is the first Chinese scale measuring caring culture for health care workers in the tertiary hospitals. It can be used to facilitate the design of a theoretical framework which explains the components of a caring culture in the Chinese hospital context. While there is evidence on the effects of culture of care on health care workers in the Western settings, very little is known about this in non‐Western cultures. The CoCB‐C is a validated measure that can be used in future investigations within the Chinese context and increases our understanding of the culture of care in hospitals.

There are limitations in this study. It recruited staff in the tertiary hospitals, which may limit the generalizability of the findings to those in other settings, such as primary or secondary hospitals, community health care centres or mental health hospitals. In addition, the test–retest reliability and predictive validity of the CoCB‐C were not conducted owing to the cross‐sectional study design and this can be considered in the design of future studies using the scale.

## 5 CONCLUSIONS

A caring culture is paramount to a positive staff and patient experience. This study indicated that the CoCB‐C is a reliable and valid instrument for the core attributes of a caring culture in the Chinese hospital context. It may result in greater communication between front‐line staff and hospital leaders about their perceptions of the work environment and then potentially lead to better job satisfaction, staff retention and patient outcomes. The CoCB‐C can be used to advance our knowledge of how the main dimensions of a caring culture manifest in health care organizations and its impact on staff and patient outcomes.

## 6 IMPLICATIONS FOR NURSING MANAGEMENT

The CoCB‐C can be used to get feedback from different groups of staff to identify areas of weakness in the culture of care, and to evaluate change over time. Tailored interventions can be designed to address the specific dimensions, and additional support can be provided to more vulnerable departments or staff groups.

## CONFLICT OF INTEREST

The authors declare that they have no known competing financial interests or personal relationships that could have appeared to influence the work reported in this paper.

## ETHICS STATEMENT

The Ethics Review Committee of King's College London granted an approval for the present study (reference number: LRS‐18/19‐11872).

## Supporting information


**Table S1** Descriptive statistics of CoCB‐C scores including floor and ceiling effects

## Data Availability

The data that support the findings of this study are available on request from the corresponding author. The data are not publicly available due to privacy or ethical restrictions.

## 1

The original English and Chinese versions of the Culture of Care Barometer.

**Table:**

English version	Chinese version
(1) I feel respected by my co‐workers	我觉得自己受到同事们的尊重
(2) I have sufficient time to do my job well	我有充足的时间来做好我的工作
(3) I have the resources I need to do a good job	我有做好工作需要的所有资源
(4) I am proud to work in this trust	在这家医院工作让我感到自豪
(5) I know who my line manager is	我知道我的直属领导是谁
(6) My line manager treats me with respect	我的直属领导尊重我
(7) The Trust values the service we provide	医院重视我们所提供的服务
(8) I would recommend this Trust as a good place to work	我会推荐他人来这家医院工作
(9) I feel well supported by my line manager	我觉得我的直属领导很支持我
(10) I am able to influence the way things are done in my team	我能够影响我们团队的工作方式
(11) I feel part of a well‐managed team	我觉得我所在的团队有着良好的管理
(12) unacceptable behaviour is consistently tackled	不可接受的行为在团队中会受到同样的处理
(13) There is strong leadership at the highest level in the Trust	医院最高层拥有强大的领导力
(14) When things get difficult, I can rely on my colleagues	遇到困难时, 我可以依靠我的同事
(15) Trust managers know how things really are	医院管理者清楚医院的实际情况
(16) I feel able to ask for help when I need it	我觉得需要时可以寻求帮助
(17) I know exactly what is expected of me in my job	我很清楚自己的工作职责
(18) I feel supported by the team to develop my potential	我得到团队的支持使我能发挥自身潜力
(19) A positive culture is visible where I work	我所在团队有积极向上的文化氛围
(20) The people I work with are friendly	与我共事的人都很友好
(21) My line manager gives me constructive feedback	我的直属领导会给我建设性的反馈意见
(22) Staff successes are celebrated by the Trust	医院会庆祝员工的成功
(23) The Trust listens to staff views	医院会倾听员工的意见
(24) I get the training and development I need	我得到了我所需的培训和发展
(25) I am able to influence the Trust how things are done in the Trust	我能够影响医院里的工作方式
(26) The Trust has a positive culture	医院具有积极向上的文化氛围
(27) I am kept well informed about what is going on in our team	我能及时获悉团队里发生的事情
(28) I have positive role models where I work	我在团队里有正面的学习榜样
(29) I feel well informed about what is happening in the Trust	我能及时获悉医院里发生的事情
(30) My concerns are taken seriously by my line manager	我的直属领导重视我关心的问题

[Correction added on 8 June 2022, after first online publication: In the Appendix, the missing Chinese characters on items 5, 10, 11, 12, 13, 18, 19, 21, 23, 26, 27, and 28 have been reinstated in this version.]

## References

[jonm13657-bib-0001] Brown, T. A. (2015). Confirmatory Factor Analysis for Applied Research. Guilford publications.

[jonm13657-bib-0002] Cai, C. Z. , Lin, Y. L. , Hu, Z. J. , & Wong, L. P. (2021). Psychological and mental health impacts of COVID‐19 pandemic on healthcare workers in China: A review. World Journal of Psychiatry, 11(7), 337–346. 10.5498/wjp.v11.i7.337 34327126 PMC8311517

[jonm13657-bib-0003] Chang, J. , & Cheng, Q. (2009). A study on evaluation index system of hospital culture. Northwest Medical Education, 17(3), 596–599.

[jonm13657-bib-0005] DeVellis, R. F. (2016). Scale Development: Theory and Applications (Vol. 26). Sage publications.

[jonm13657-bib-0006] Gao, X. , Yan, S. , Wu, W. , Zhang, R. , Lu, Y. , & Xiao, S. (2019). Implications from China patient safety incidents reporting system. Therapeutics and Clinical Risk Management, 15, 259–267. 10.2147/TCRM.S190117 30799925 PMC6371930

[jonm13657-bib-0007] Gonzalez, R. , & Griffin, D. (2001). Testing parameters in structural equation modeling: Every “one” matters. Psychological Methods, 6(3), 258–269. 10.1037/1082-989X.6.3.258 11570231

[jonm13657-bib-0008] Hesselink, G. , Kuis, E. , Pijnenburg, M. , & Wollersheim, H. (2013). Measuring a caring culture in hospitals: A systematic review of instruments. BMJ Open, 3(9), e003416. 10.1136/bmjopen-2013-003416 PMC3787470 24065697

[jonm13657-bib-0009] Hingorjo, M. R. , & Jaleel, F. (2012). Analysis of one‐best MCQs: The difficulty index, discrimination index and distractor efficiency. The Journal of the Pakistan Medical Association, 62(2), 142–147. 22755376

[jonm13657-bib-0004] IBM Corp . (2016). IBM SPSS Statistics for Windows, Version 24.0. IBM Corp.

[jonm13657-bib-0010] Jia, X. L. , Zhou, H. Z. , Zhao, Y. , Zheng, L. , Wei, Q. , & Zheng, X . (2014). Investigation on hospital violence during 2003 to 2012 in China. Chinese Hospitals, 18(3), 1–3.Chinese.

[jonm13657-bib-0011] Jiang, F. , Hu, L. , Rakofsky, J. , Liu, T. , Wu, S. , Zhao, P. , Hu, G. , Wan, X. , Liu, H. , Liu, Y. , & Tang, Y. L. (2018). Sociodemographic characteristics and job satisfaction of psychiatrists in China: Results from the first nationwide survey. Psychiatric Services, 69(12), 1245–1251. 10.1176/appi.ps.201800197 30301449

[jonm13657-bib-0012] Lai, J. , Ma, S. , Wang, Y. , Cai, Z. , Hu, J. , Wei, N. , Wu, J. , Du, H. , Chen, T. , Li, R. , Tan, H. , Kang, L. , Yao, L. , Huang, M. , Wang, H. , Wang, G. , Liu, Z. , & Hu, S. (2020). Factors associated with mental health outcomes among health care workers exposed to coronavirus disease 2019. JAMA Network Open, 3(3), e203976. 10.1001/jamanetworkopen.2020.3976 32202646 PMC7090843

[jonm13657-bib-0013] Liu, J. , Yu, W. , Ding, T. , Li, M. , & Zhang, L. (2019). Cross‐sectional survey on job satisfaction and its associated factors among doctors in tertiary public hospitals in Shanghai, China. BMJ Open, 9(3), e023823. 10.1136/bmjopen-2018-023823 PMC6429855 30826758

[jonm13657-bib-0014] Liu, J. , Zheng, J. , Liu, K. , Liu, X. , Wu, Y. , Wang, J. , & You, L. (2019). Workplace violence against nurses, job satisfaction, burnout, and patient safety in Chinese hospitals. Nursing Outlook, 67(5), 558–566. 10.1016/j.outlook.2019.04.006 31202444

[jonm13657-bib-0015] Luthans, F. , Norman, S. M. , Avolio, B. J. , & Avey, J. B. (2008). The mediating role of psychological capital in the supportive organizational climate—Employee performance relationship. Journal of Organizational Behavior, 29(2), 219–238. 10.1002/job.507

[jonm13657-bib-0016] Molassiotis, A. , Coventry, P. A. , Stricker, C. T. , Clements, C. , Eaby, B. , Velders, L. , Rittenberg, C. , & Gralla, R. J. (2007). Validation and psychometric assessment of a short clinical scale to measure chemotherapy‐induced nausea and vomiting: The MASCC antiemesis tool. Journal of Pain and Symptom Management, 34(2), 148–159. 10.1016/j.jpainsymman.2006.10.018 17509816

[jonm13657-bib-0017] Polit, D. F. , Beck, C. T. , & Owen, S. V. (2007). Is the CVI an acceptable indicator of content validity? Appraisal and recommendations. Research in Nursing & Health, 30(4), 459–467. 10.1002/nur.20199 17654487

[jonm13657-bib-0018] Powell, M. , Dawson, J. , Topakas, A. , Durose, J. , & Fewtrell, C. (2014). Staff satisfaction and organisational performance: Evidence from a longitudinal secondary analysis of the NHS staff survey and outcome data. Health Services and Delivery Research, 2(50), 1–306. 10.3310/hsdr02500 25642572

[jonm13657-bib-0019] Rafferty, A. M. , Philippou, J. , Fitzpatrick, J. M. , & Ball, J. (2015). Culture of Care Barometer: Report to NHS England on the Development and Validation of an Instrument to Measure ‘Culture of Carein NHS Trusts’. King's College London.

[jonm13657-bib-0020] Rafferty, A. M. , Philippou, J. , Fitzpatrick, J. M. , Pike, G. , & Ball, J. (2017). Development and testing of the 'Culture of care Barometer' (CoCB) in healthcare organisations: A mixed methods study. BMJ Open, 7(8), e016677. 10.1136/bmjopen-2017-016677 PMC5629717 28821526

[jonm13657-bib-0021] Rosseel, Y. (2012). Lavaan: An R package for structural equation modeling. Journal of Statistical Software, 48, 1–36. 10.18637/jss.v048.i02

[jonm13657-bib-0022] Rytterstrom, P. , Unosson, M. , & Arman, M. (2013). Care culture as a meaning‐making process: A study of a mistreatment investigation. Qualitative Health Research, 23(9), 1179–1187. 10.1177/1049732312470760 23264536

[jonm13657-bib-0023] Salmela, S. , Koskinen, C. , & Eriksson, K. (2017). Nurse leaders as managers of ethically sustainable caring cultures. Journal of Advanced Nursing, 73(4), 871–882. 10.1111/jan.13184 27732746

[jonm13657-bib-0024] Sousa, V. D. , & Rojjanasrirat, W. (2011). Translation, adaptation and validation of instruments or scales for use in cross‐cultural health care research: A clear and user‐friendly guideline. Journal of Evaluation in Clinical Practice, 17(2), 268–274. 10.1111/j.1365-2753.2010.01434.x 20874835

[jonm13657-bib-0025] Sun, J. , Zhang, L. , Sun, R. , Jiang, Y. , Chen, X. , He, C. , & Wei, J. (2018). Exploring the influence of resiliency on physician trust in patients: An empirical study of Chinese incidents. PLoS ONE, 13(12), e0207394. 10.1371/journal.pone.0207394 30540768 PMC6291099

[jonm13657-bib-0026] Terwee, C. B. , Bot, S. D. , de Boer, M. R. , van der Windt, D. A. , Knol, D. L. , Dekker, J. , Bouter, L. M. , & de Vet, H. C. (2007). Quality criteria were proposed for measurement properties of health status questionnaires. Journal of Clinical Epidemiology, 60(1), 34–42. 10.1016/j.jclinepi.2006.03.012 17161752

[jonm13657-bib-0027] Voronov, M. , & Singer, J. A. (2002). The myth of individualism‐collectivism: A critical review. The Journal of Social Psychology, 142(4), 461–480. 10.1080/00224540209603912 12153123

[jonm13657-bib-0028] Wan, Q. , Li, Z. , Zhou, W. , & Shang, S. (2018). Effects of work environment and job characteristics on the turnover intention of experienced nurses: The mediating role of work engagement. Journal of Advanced Nursing, 74(6), 1332–1341. 10.1111/jan.13528 29350781

[jonm13657-bib-0029] Wei, H. , Corbett, R. W. , Ray, J. , & Wei, T. L. (2019). A culture of caring: The essence of healthcare interprofessional collaboration. Journal of Interprofessional Care, 1–8. 10.1080/13561820.2019.1641476 31390903

[jonm13657-bib-0030] Weiss, D. J. , Dawis, R. V. , & England, G. W. (1967). Manual for the Minnesota satisfaction questionnaire. In Minnesota Studies in Vocational Rehabilitation.

[jonm13657-bib-0031] Xi, X. , Xu, H. , Zhang, J. , Zhang, X. , Zhang, Z. , & Shi, J . (2016). Analysis of hospital cultures impact on employee satisfaction of a tertiary hospital. Chinese Journal of Hospital Administration 32(2), 145–148. Chinese.

[jonm13657-bib-0032] Zhan, Y. , Ma, S. , Jian, X. , Cao, Y. , & Zhan, X. (2020). The current situation and influencing factors of job stress among frontline nurses assisting in Wuhan in fighting COVID‐19. Frontiers in Public Health, 8, 579866. 10.3389/fpubh.2020.579866 33194981 PMC7649821

[jonm13657-bib-0033] Zhu, J. , Stone, T. , & Petrini, M. (2021). The ethics of refusing to care for patients during the coronavirus pandemic: A Chinese perspective. Nursing Inquiry, 28(1), e12380. 10.1111/nin.12380 32955787 PMC7537035

