# Supplementary material for: Front‐line staff perspectives on a caring culture in Chinese hospitals: Validation of a Chinese version of the Culture of Care Barometer
Source: J Nurs Manag. 2022 May 31;30(6):2093–102. doi: 10.1111/jonm.13657 (PMC9796908; doi:10.1111/jonm.13657)
Supplement: Supplementary file 1 — Table S1 Descriptive statistics of CoCB‐C scores including floor and ceiling effects [file JONM-30-2093-s001.docx]

**Table S1** Descriptive statistics of CoCB-C scores including floor and ceiling effects

| Outcome Measure | Scores |  |  |  |  | Floor |  |  | Ceiling |  |
| --- | --- | --- | --- | --- | --- | --- | --- | --- | --- | --- |
|  | Score Range | n | Mean ± SD | Median |  | n | % |  | n | % |
| CoCB-C | 30-150 | 2365 | 127.0 ±18.24 | 124 |  | 0 | 0 |  | 320 | 13.5 |
